# Changing Radiotherapy Paradigms in Penile Cancer

**DOI:** 10.1016/j.euros.2021.12.005

**Published:** 2022-01-04

**Authors:** Peter A.S. Johnstone, Philippe E. Spiess, Geoff Sedor, G. Daniel Grass, Kosj Yamoah, Jacob G. Scott, Javier F. Torres-Roca

**Affiliations:** a Moffitt Cancer Center and Research Institute, Tampa, FL, USA; b Cleveland Clinic, Cleveland, OH, USA

**Keywords:** Penile cancer, Radiation sensitivity, Radiation

## Abstract

Radiation therapy (RT) has not been prominent in the treatment of penile cancer because of poorly reproducible results when used in the adjuvant setting. A genomic signature has recently been described that assays radiosensitivity of tumors and informs radiotherapy doses in these cases. Clinical validation in more than 1600 patients demonstrated associations with both overall survival and time to first recurrence. In addition, the signature predicted and quantified the therapeutic benefit of RT for each individual patient. Since penile cancer patients were not part of this analysis, we applied the model to patients with primary and nodal penile cancer tissue and clinical outcomes.

***Patient summary***: Radiotherapy has not been widely used for treatment of penile cancer. New genetic data suggest that radiation doses commonly used to treat penile cancer are too low. This would explain prior poor results using radiation in this disease.

For decades, a radiotherapy (RT) dose of 50 Gy has been considered sufficient to control >90% of microscopic disease in squamous cell carcinoma of the head and neck and in breast adenocarcinoma, and was widely used for other disease sites. However, we have found that dose to be ineffective clinically when used for patients with squamous cell carcinoma of the penis (PeSCC) postoperatively. Defining a more personalized and tailored RT strategy is critically important since prior European Association of Urology guidelines advocated against the use of perioperative RT in patients with adverse clinicopathological features following inguinal lymph node dissection for node-positive PeSCC [1].

We developed a multigene signature, the Radiosensitivity Index (RSI), to estimate the intrinsic radiosensitivity of solid tumors irrespective of origin. RSI is a Clinical Laboratory Improvement Amendments (CLIA)-certified assay. A further elaboration, the Genomic-Adjusted Radiation Dose (GARD), relates the biologic effect of RT dose by integrating the RSI into a linear quadratic model [2]. Across cancer types, GARD is significantly predictive of overall survival and time to first recurrence in patients treated with conventionally fractionated RT, while the physical RT dose is not [3]. These results verify the concept in varied clinical cohorts across cancer types.

In this analysis, we sampled 25 primary and 17 nodal PeSCC samples for RSI score (range 0–1.0). Median RSI was 0.48 (range 0.215–0.682) for the primary tumor cohort [4] and 0.40 (range 0.24–0.67) for the nodal tissue cohort [5]. This RSI for PeSCC is quite similar to the RSI for melanoma, one of the most radioresistant lesions [4]. We modeled GARD using a dose of 50 Gy for the primary PeSCC samples, with scores ranging from 9.56 to 38.39 (median 18.25), implying a variable therapeutic effect with RT at this uniform dose level. Using a cutpoint at the median GARD score, we predicted that local control would be achieved in 52% of PeSCC lesions after 50 Gy, which increased to 84% when GARD was modeled at 66 Gy. In an independent cohort of 34 patients treated with adjuvant RT (median follow-up 1 yr), the local control rate observed was 59% [4]. Thus, the traditional RT doses used might simply have been too low, and implementation of GARD has the potential to improve tumor control outcomes by approximately 20–30% for the inguinal regions postoperatively. Using our data, Fig. 1 reveals that patients with primary lesions with a GARD score >20 (more radiosensitive) had better survival than those with a GARD score <20 (more radioresisitant); the two cohorts are not significantly different because of small numbers.

**Fig. 1:**
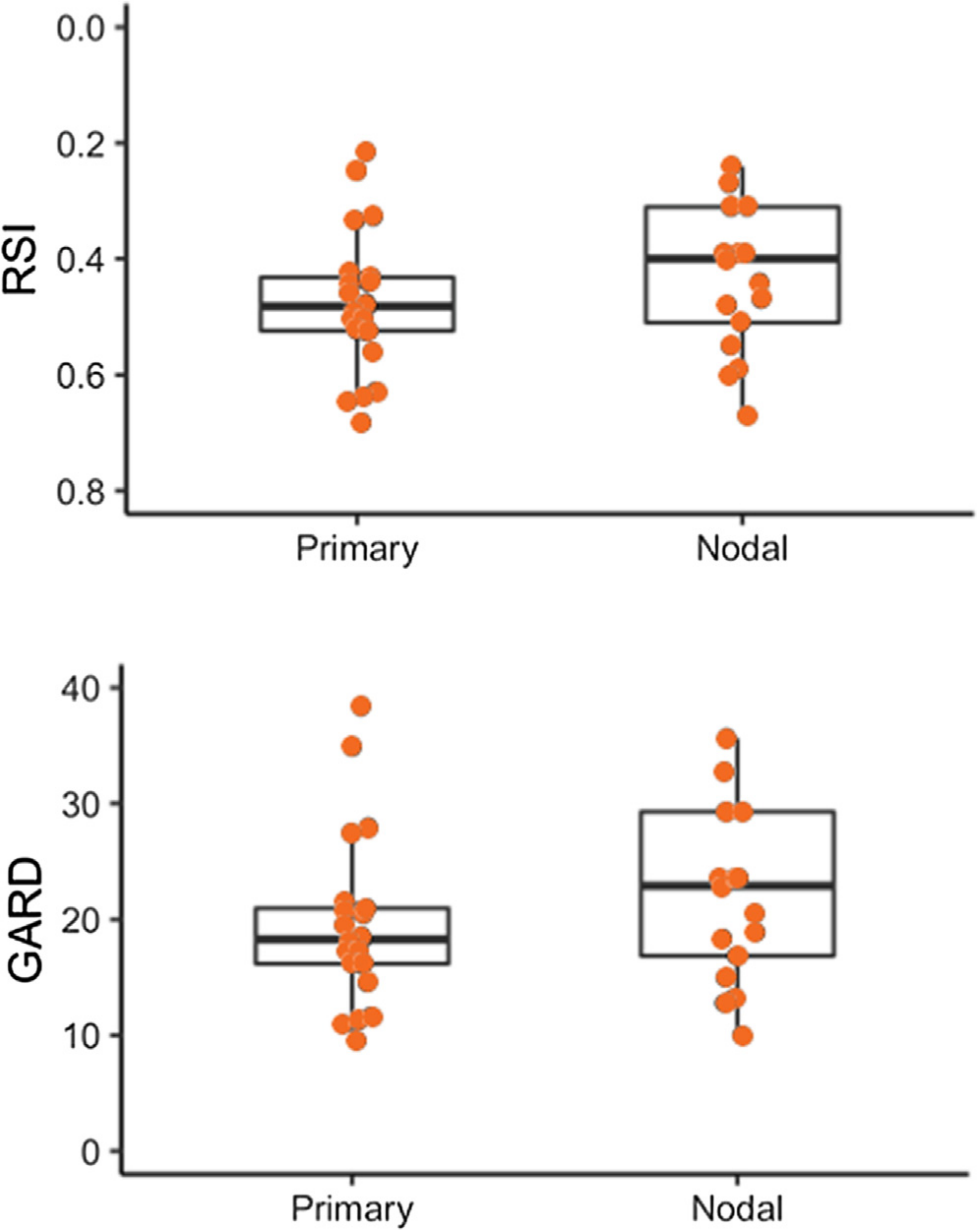
Distribution of Radiosensitivity Index (RSI) and Genomic-Adjusted Radiation Dose (GARD) scores in the cohort, separated by primary or nodal origin. GARD was calculated using standard-of-care dosing of 50 Gy. As GARD increases with decreasing RSI, the *y* axis for the top row is inverted to preserve the underlying distributions represented*.*

RT doses have never been tailored to individual patient tumor biology, increasing the likelihood of overtreatment and undertreatment. We propose that RSI and GARD are measurable tumor features that may be used as tools to optimize the therapeutic ratio in PeSCC. Such a personalized approach for RT is overdue and collaborative international trials are being planned to assess the efficacy of GARD in dose personalization. Once the ongoing InPACT trial has completed accrual, our next proposal would be an RSI/GARD-informed personalized dose trial.

  ***Author contributions***: Peter A.S. Johnstone had full access to all the data in the study and takes responsibility for the integrity of the data and the accuracy of the data analysis.

*Study concept and design*: Johnstone.

*Acquisition of data*: Johnstone, Torres-Roca.

*Analysis and interpretation of data*: Scott, Sedor, Torres-Roca.

*Drafting of the manuscript*: Grass, Spiess, Yamoah, Johnstone.

*Critical revision of the manuscript for important intellectual content*: Grass, Johnstone, Spiess, Sedor, Scott, Yamoah, Torres-Roca.

*Statistical analysis*: Scott, Sedor.

*Obtaining funding*: None.

*Administrative, technical, or material support*: Sedor, Spiess, Johnstone.

*Supervision*: Spiess, Johnstone, Scott, Torres-Roca.

*Other*: None.

  ***Financial disclosures:*** Peter A.S. Johnstone certifies that all conflicts of interest, including specific financial interests and relationships and affiliations relevant to the subject matter or materials discussed in the manuscript (eg, employment/affiliation, grants or funding, consultancies, honoraria, stock ownership or options, expert testimony, royalties, or patents filed, received, or pending), are the following: Philippe E. Spiess is vice-chair of the NCCN bladder and penile cancer panel, president of the Global Society of Rare Genitourinary Tumors, and a member of the ASCO/EAU panel on penile cancer. Jacob G. Scott and Javier F. Torres-Roca hold intellectual property in RSI, GARD, and a prescription dose based on RSI (known as RxRSI) and equity in Cvergenx, a company that seeks to commercialize these methods. Patents held by Moffitt Cancer Center are as follows: RSI (patent numbers 7 879 545, 8 655 598, 8 660 801, and 9 846 762); GARD (patent number 10 697 023); and Cvergenx (RxRSI, pending application number 16/658 961). Javier F. Torres-Roca is a cofounder and board member of Cvergenx. The remaining authors have nothing to disclose.

  ***Funding/Support and role of the sponsor:*** None.
